# Feasibility and Acceptability of Clinical Pediatric Telerehabilitation Services

**DOI:** 10.5195/ijt.2020.6336

**Published:** 2020-12-08

**Authors:** Kelly Tanner, Rachel Bican, Jamie Boster, Catie Christensen, Candace Coffman, Kristin Fallieras, Rene Long, Christine Mansfield, Sara O'ROURKE, Lindsey Pauline, Grace Sagester, James Marrie

**Affiliations:** 1 Division of Clinical Therapies, Nationwide Children's Hospital, Columbus, Ohio, USA

**Keywords:** Healthcare, Patient-centered care, Pediatric rehabilitation, Telerehabilitation

## Abstract

**Objective:**

Telerehabilitation has long been recognized as a promising means of providing pediatric services; however, significant barriers such as cost, payor reimbursement, and access prevented widespread use. The advent of the COVID-19 pandemic necessitated rapid adoption of telerehabilitation into clinical practice to provide access to care while maintaining social distancing. The purpose of this study is to present clinical data on the feasibility and acceptability of speech-language pathology, developmental occupational and physical therapies, and sports and orthopedic therapies telerehabilitation delivered in a pediatric hospital setting.

**Methods:**

Telerehabilitation services were rapidly implemented in three stages: building the foundation, implementing, and refining this service delivery model. Paper patient satisfaction surveys were administered as part of ongoing quality improvement efforts throughout 2019 and were adapted for online administration in 2020 for telerehabilitation patients. Outpatient visit counts by type (in-person, phone, and video) were extracted from the electronic medical record using data warehousing techniques.

**Results:**

Historical patient satisfaction rates from 2019 indicated high patient satisfaction (98.97% positive responses); these results were maintained for telerehabilitation visits (97.73%), indicating that families found telerehabilitation services acceptable. Patient volume returned to 73.5% of pre-pandemic volume after the implementation of telerehabilitation services.

**Conclusions:**

Pediatric telerehabilitation is feasible to provide in clinical settings, and the services are acceptable to patient families. Future work is needed to evaluate the impact of telerehabilitation services on patient care and applications for ongoing use of this delivery model.

Telerehabilitation has been proposed as a potentially effective means of providing pediatric services (Olson et al., 2018; Shigekawa et al., 2018; Tenforde et al., 2017). Telerehabilitation, also referred to more broadly as telepractice or telehealth, has been found to be effective, efficient, affordable, (Olson et al., 2018) and generally equivalent to in-person care (Shigekawa et al., 2018). While the effectiveness and applicability of this service delivery model may vary by pediatric specialty, setting, and patient preference (Tomines, 2019), it can be used for many purposes including delivery of care, education for patients and families, and conducting research (Burke et al., 2015; Utidjian & Abramson, 2016). Use of telehealth and telerehabilitation has been especially important in responding to emergencies and disasters to provide undisrupted access to pediatric care (Burke et al., 2015). Implementation of telehealth services has increased with recent advances in communication technology such as the proliferation of video-based platforms, access to high-speed internet, and higher consumer demand (Burke et al., 2015; Tomines, 2019). Telerehabilitation has been offered as a potential solution to barriers to providing care.

Telerehabilitation has been used by a variety of fields and with many different patient populations. This service delivery model has been shown to be effective in the orthopedics (Lee et al., 2018), neurology (Tenforde et al., 2017), and mental health settings (Douglas et al., 2014; Gloff et al., 2015; Nelson & Sharp, 2016; Tomines, 2019). Professional organizations including the American Speech Language Hearing Association (ASHA), American Occupational Therapy Association (AOTA), and American Physical Therapy Association (APTA) have all published statements in support of telerehabilitation use in practice (American Occupational Therapy Association, 2018; American Physical Therapy Association, 2019; American Speech-Language-Hearing Association, 2020). In speech-language pathology, researchers have suggested that telerehabilitation can support the assessment and treatment of articulation disorders (Crutchley & Campbell, 2010), language and cognitive disorders (Brennan et al., 2004; Weidner & Lowman, 2020), aphasia (Hall et al., 2013), autism spectrum disorder (ASD; Sutherland et al., 2018) and dysarthria (Hill et al., 2006). There is additional research to support telerehabilitation interventions for fluency disorders (McGill et al., 2018), dysphagia (Coyle, 2012), and voice disorders (Rangarathnam et al., 2016). For occupational therapy, telerehabilitation has long been touted as an emerging, promising area of practice that may alleviate problems such as patient access, cost of services, and allow practitioners to intervene within the natural environment (Cason, 2014; Eckberg Zylstra, 2013). Telerehabilitation technologies have been shown to be feasible and effective in the context of pediatric occupational therapy studies for children with cerebral palsy (CP; Reifenberg et al., 2017) and ASD (Little et al., 2018). It has also been shown to be a valid and reliable intervention for musculoskeletal physical therapy conditions (Lee et al., 2018). Intervention studies comparing in-person to telerehabilitation physical therapy demonstrated similar improvements in health outcomes including pain and function (Lovo Grona et al., 2016). In physical therapy for developmental disorders, telerehabilitation has been used as an alternative to in-person treatment with success (Olson et al., 2018).

Despite advocacy from researchers and professional organizations, pediatric telerehabilitation has not been widely implemented in clinical settings. Thus, there is a *gap in the literature* regarding the feasibility and acceptability of widespread, clinical pediatric telerehabilitation services. The primary limiting factors for implementation have been the limited payor reimbursement, perceived and actual technological barriers, liability concerns, and privacy concerns (Brophy, 2017; Dorsey & Topol, 2016; Lee et al., 2018; Olson et al., 2018; Sauers-Ford et al., 2019; Tomines, 2019). The COVID-19 pandemic resulted in a lessening of the aforementioned barriers to clinical implementation, as well as presented an urgent need to provide safe and effective rehabilitation services to patients during a vulnerable time (Badawy & Radovic, 2020; Ben-Pazi et al., 2020; Ohannessian et al., 2020; Olayiwola et al., 2020). While the pandemic has had many negative effects on health and well-being of people around the world, it has resulted in an unprecedented rise in the use of telerehabilitation out of necessity for providing safe access to care during a public health crisis.

Our hospital division rapidly implemented telerehabilitation services for several of our outpatient pediatric departments, including: Speech Pathology, Developmental Occupational Therapy (OT) and Physical Therapy (PT), and Sports and Orthopedic Therapies. The objective of this paper is to describe the feasibility and acceptability of pediatric telerehabilitation, which can result in continued access to care for patients while maintaining high levels of patient satisfaction.

## METHODS

### SETTING

This study took place in a large, free-standing pediatric hospital in the Midwest that draws patients from urban, suburban, and rural locations. The Division of Clinical Therapies includes multiple outpatient therapy departments employing a total of 221 full-time equivalents in both clinical and non-clinical staff members. Across all of these outpatient departments, 4,500 patients per week were seen for outpatient therapy visits prior to the COVID-19 pandemic, across 24 outpatient buildings. Although research on telerehabilitation service delivery had previously been completed within the Speech Pathology Department, no clinical telerehabilitation services were offered prior to the COVID-19 pandemic.

### IMPLEMENTATION OF TELEREHABILITATION

**Figure 1:**
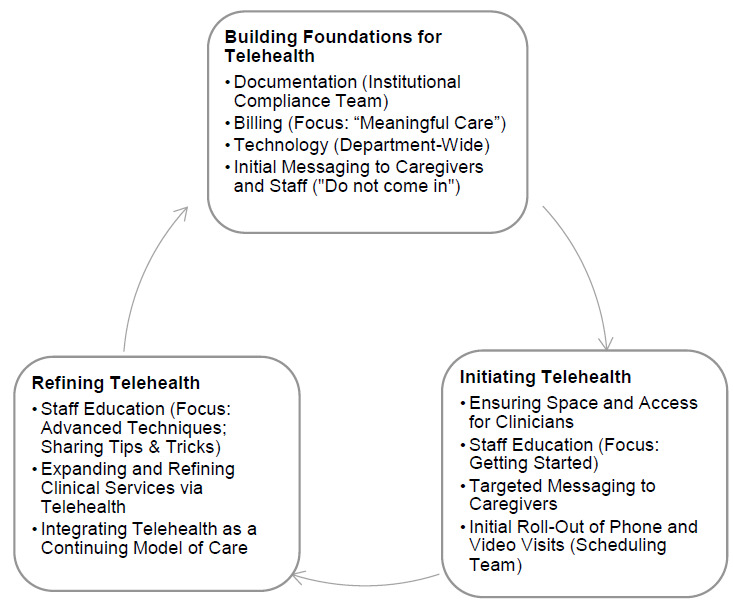
Model for Rapid Clinical Implementation of Telerehabilitation that Includes Three Phases: Building Foundations for Telehealth, Initiating Telehealth, and Refining Telehealth

Rapid implementation of telerehabilitation included several key components, presented in Figure 1. All departments progressed through the three general stages of Building Foundations, Initiating, and Refining, although the order of progression varied by department according to staffing patterns, patient volume, and whether they were the first to adopt telerehabilitation. The Speech Pathology Department quickly changed 100% of visits from in-person to telerehabilitation visits one day after the governor's stay-at-home order was issued in order to create social distancing options within our clinics and waiting rooms. The Sports and Orthopedic Therapies Department quickly saw a reduction in in-person visits due to multiple factors, including decreased pediatric involvement in sports based on the stay-at-home order, a decline in surgeries based on the halting of elective surgeries, and a general reduction in “non-essential” hospital visits; this department was able to implement telerehabilitation services starting in the week after the stay-at-home order was issued. The Developmental OT and PT Department also followed a thoughtful approach to initiating telerehabilitation, beginning their first telerehabilitation visits two weeks after the initial onset of visits completed by the Speech Pathology Department. Both departments continued to see a small amount of “essential” patients in-person, and the Developmental OT and PT Department also utilized a hybrid model in which patients could be seen for both in-person and telerehabilitation visits throughout an episode of care.

### BUILDING FOUNDATIONS FOR TELEREHABILITATION

The first stage of rapid telerehabilitation implementation was building the foundations for telerehabilitation, specifically implementing structural supports needed for efficient and sustainable telerehabilitation practices. The Developmental OT and PT Department and the Sports and Orthopedic Therapies Department started in this phase, while the Speech Pathology Department completed many of the steps within this phase after first adopting telerehabilitation as a delivery model.

#### DOCUMENTATION

Documentation within the electronic medical record (EMR) was important to capture the services provided and to ensure compliance with regulatory bodies and payors. The Institutional Compliance Team provided support for all departments with templates for documentation created and adapted for each department's needs.

#### BILLING

The focus of each department during this time of rapid implementation was on providing meaningful, high-quality care to all patients. The transition to telerehabilitation at our institution occurred prior to approved payor reimbursement for these services. Our telerehabilitation services followed all payor regulations in terms of documentation and the same level of care was provided to our patients. Throughout the telerehabilitation implementation process, it became important to track developments in reimbursement; of note, our state Medicaid program and most private insurance providers did ultimately cover telerehabilitation services.

#### TECHNOLOGY

Ensuring adequate technology was a key component to providing meaningful telerehabilitation services. Initially, most departments only had access to phone calls for providing telerehabilitation services. Within the first days and weeks, however, all departments gained access to a secure video platform (Zoom; *Zoom*, 2020) which was integrated into the EMR. Telerehabilitation phone visits continued to be offered as a solution for families who did not have access to a device compatible with Zoom or declined use of Zoom for another reason.

#### INITIAL MESSAGING TO CAREGIVERS AND STAFF

The most important initial messaging to caregivers was to communicate when their child's visits would be converted to telerehabilitation and to provide instructions on how to access telerehabilitation services. Staff also received early communication regarding when telerehabilitation services would be initiated for their department.

### INITIATING TELEREHABILITATION

Once the foundation was established for rapid implementation of telerehabilitation, departments began the second stage to actively implement telerehabilitation pediatric rehabilitation with the following considerations.

#### ENSURING SPACE AND ACCESS FOR CLINICIANS

While institutional supports were being put into place, individual departments had to ensure that all clinicians had adequate space to promote social distancing within staff offices, and access to the technology they needed to provide care. Some computers were shared prior to the initiation of telerehabilitation services and staff offices were busy, often crowded places. New computers and software were ordered for those who needed them and new spaces (treatment rooms, research rooms, vacant offices) were identified for staff to work. Several departments also allowed staff to work from home on specific days in order to further promote social distancing.

#### STAFF EDUCATION

Staff education on the provision of telerehabilitation services was provided using a multi-tiered approach including structured education and more informal, discussion-based opportunities. The focus of education during this phase was providing staff with enough information to feel confident to begin providing meaningful telerehabilitation services to their patients. Webinars on evidence-based practices and telerehabilitation-focused resources were created by an emergency task force of clinicians, research coordinators, evidence-based practice coordinators, and quality improvement team members. We leveraged existing systems for sharing documents, convening meetings, and structuring documents.

#### TARGETED MESSAGING TO CAREGIVERS

While caregivers were initially told not to come to their hospital location for their visits, further messaging was later provided regarding telerehabilitation services. We created a script for therapists to answer common questions regarding the efficacy of telerehabilitation visits and what to expect during their appointment. In addition, the Onsite Scheduling Team contacted each currently scheduled patient and enrolled the family in the EMR patient portal, confirmed their first telerehabilitation visit, and walked the family through technology troubleshooting to make the first visit successful. Each contact by the Onsite Scheduling Team required 15-30 minutes of dedicated time, with many encounters walking the family through the process for accessing Zoom on their device, outlining all expectations, and answering questions for the upcoming visit.

#### INITIAL ROLL-OUT OF PHONE AND VIDEO VISITS

The Onsite Scheduling Team established themselves as a key component in an effective telerehabilitation effort when rolling out both phone and video telerehabilitation visits. Before the COVID-19 pandemic, the Central Scheduling Department scheduled all therapy evaluations, with a 10-person Onsite Scheduling Team providing support for scheduling treatments for the Speech Pathology and Developmental OT and PT Departments only. At the start of the pandemic, the Onsite Scheduling Team began delving into every aspect of scheduling, registration, and support of telerehabilitation.

Staff education also took place to ensure that clinicians were comfortable using the available technology. This occurred through scheduled webinars, email communication, and one-on-one training with staff members as needed.

### REFINING TELEREHABILITATION

Once telerehabilitation services were established, each department continued into the third stage of rapid implementation and began to refine their services in a variety of ways.

#### 

##### STAFF EDUCATION

While the initial focus of staff education was “getting started” in telerehabilitation, later educational sessions focused on more advanced treatment techniques and completing evaluations via telerehabilitation. Due to the paucity of scientific literature available on telerehabilitation at this time, for this phase of education we relied on the other two pillars of evidence-based practice to guide our clinical reasoning: clinical expertise and stakeholder preferences (Sackett et al., 1996). We did this by highlighting the “Tips & Tricks” of staff identified as telerehabilitation champions. These included tips on patient positioning, working effectively with parents, creative uses of technology, and specific treatment ideas such as websites and other interactive, virtual activities.

##### EXPANDING AND REFINING CLINICAL SERVICES VIA TELEREHABILITATION

Once initial clinical services were established via telerehabilitation, some departments began offering more specialized clinical services, while others focused on refining their services. For example, the Developmental OT and PT Department initially offered only treatment via telerehabilitation, but soon began offering evaluations as well. The Speech Pathology Department offered most types of evaluations from the beginning, but started incorporating augmentative and alternative communication (AAC) evaluations several weeks later. The Sports and Orthopedic Therapies Department refined which patients were being seen via telerehabilitation, as they continued to evaluate which patients could safely be seen in-person based on the evolving nature of the pandemic.

#### INTEGRATING TELEREHABILITATION AS A CONTINUING MODEL OF CARE

As the pandemic continues and in-person visits are being utilized more, we are considering how telerehabilitation may be integrated into our departments as a continuing model of care. We are also advocating to hospital leaders and legislative officials for the continued use of telerehabilitation, so that it may be a sustainable model of care in the future to improve access to care and mitigate risk for vulnerable patients.

### DATA COLLECTION PROCEDURES & ANALYSIS

All data were collected as part of ongoing quality improvement efforts in the division. This study was reviewed by the Institutional Review Board and a waiver of consent was granted due to the retrospective nature of the study.

#### 

##### PATIENT VISIT COUNTS BY TYPE

Patient visit counts by type (i.e., in-person, video telerehabilitation, or phone telerehabilitation) for all outpatient visits during Weeks 6-26 of the 2020 calendar year were collected via retrospective chart review of the hospital's EMR using data warehousing technology. This date range was selected to provide several weeks of baseline data prior to the COVID-19 pandemic, during the governor's stay-at-home order, and several weeks of data after the stay-at-home order was lifted. Data were analyzed in Microsoft Excel using descriptive statistics.

#### PATIENT SATISFACTION

Prior to the COVID-19 pandemic, an 11-item, Likert scale patient satisfaction survey was administered to parents/caregivers throughout the 2019 calendar year. Surveys were distributed for a 1-week period each quarter at all ambulatory locations as part of ongoing quality improvement efforts. Each parent/caregiver received the paper survey at registration and was asked to complete the survey by the end of their visit. Families receiving care from multiple disciplines at that visit were only asked to complete one survey for the day. Survey results were compiled based on ambulatory location. Results were summarized in Microsoft Excel tables by site, collated to provide historical context, and analyzed using descriptive statistics.

During the COVID-19 pandemic, slightly different survey methods were adopted. To maintain consistency with pre-pandemic survey protocol, a 1-week period was identified for surveys to be distributed to parents/caregivers. Post-telerehabilitation visit completion, clinicians sent an 8-item, Likert scale REDCap survey via a secure electronic messaging system linked to the EMR to the parent/caregiver for completion. The pre-COVID survey questions were modified to capture the telerehabilitation experience. Survey results were compiled for all disciplines within the division of clinical therapies. Results were collected in REDCap and exported to Microsoft Excel for analysis using descriptive statistics.

## RESULTS

### FEASIBILITY

**Figure 2:**
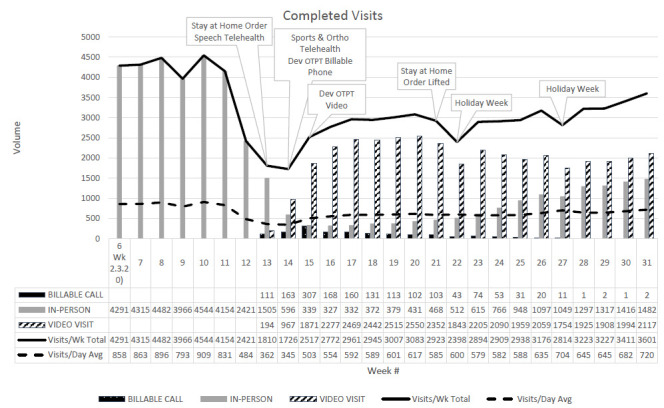
Completed Visit Types for All Departments Over a 26-week Period (2/3/2020-7/31/2020)

*Note.* Speech= Speech Pathology Department; Sports & Ortho= Sports and Orthopedic Therapies Department; Dev OTPT= Developmental OT and PT Department

Provision of video and phone visits across time indicates that delivery of telerehabilitation services is feasible in a clinical setting (Figure 2). The marked decline in in-person visits during Week 12 of the 2020 calendar year reflects growing coverage of the pandemic in the news and increased hesitancy for patients to attend in-person visits. In Week 13, the governor issued a stay-at-home order for the state and the Speech Pathology Department initiated telerehabilitation services. The Sports and Orthopedic Therapies Department initiated telerehabilitation services in Week 14 and the Developmental OT and PT Department initiated services in week 15. The governor lifted the stay-at-home order in week 21, but telerehabilitation services continued to be utilized to promote continued social distancing recommendations. Week 22 contained a holiday (Memorial Day) during which outpatient services were not offered resulting in a drop in total visits per week; however, the average number of visits per day remained stable. By Week 26, the total number of visits (3176) represented 73.5% of the weekly average visits for pre-pandemic Weeks 6-10 (4319.6).

## ACCEPTABILITY

For the 2019 calendar year (i.e., prior to the COVID-19 pandemic), average patient satisfaction survey responses were 98.97% positive (i.e., “Strongly Agree” or “Agree”) across questions for all outpatient departments and all ambulatory sites.

The results of patient satisfaction surveys completed by those who received telerehabilitation services during the COVID-19 pandemic indicates that parents of patients found their services to be acceptable (Table 1). The word “telehealth” was used throughout the survey for consistency with other similar surveys in our institution. For each question, over 95% of respondents indicated a positive response of either “Strongly Agree” or “Agree,” with an average positive response rate of 97.73% across questions. The lowest percentage of positive responses was related to technology use: “It was easy to use the Zoom video conferencing tool” (95.5% positive). The highest percentage of positive responses were related to interaction with the therapist: “The therapist demonstrated respect, friendliness, and professionalism” (98.95%); “The therapist prepared me for my telehealth appointment” (98.16%); “I understood the outcome of my telehealth appointment and the next steps” (98.54%).

**Table 1:** Telerehabilitation Patient Satisfaction Survey Results (n=767)

Survey statement	Number of responses	% Positive responses
It was easy to use the Zoom video conferencing tool.	761	95.53%
The therapist demonstrated respect, friendliness, and professionalism.	762	98.95%
The length of time needed for this telehealth appointment met my expectations.	761	96.98%
The therapist prepared me for my telehealth appointment.	759	98.16%
The therapist answered all of my questions during the telehealth appointment.	757	98.94%
I understood the outcome of my telehealth appointment and the next steps.	755	98.54%
My overall experience with [hospital name] telehealth was positive.	757	97.09%
Overall, my needs were met and I would recommend others to [hospital name]	762	97.64%

## DISCUSSION

The results of this study indicate that pediatric clinical telerehabilitation services are feasible and acceptable to families. This is in line with previous research that supports the feasibility of telerehabilitation, including the positive experiences of families whose children received care through this model (Little et al., 2018; Shigekawa et al., 2018; Tomines, 2019). There can be important benefits to the use of telerehabilitation, including improved access to services, improved access to specific providers or specialists, and prevention of unnecessary delays in receiving care (Cason, 2014; Olson et al., 2018; Utidjian & Abramson, 2016). During the early weeks and months of the COVID-19 pandemic, telerehabilitation provided a safe, accessible model of care that limited trips to hospital locations and promoted social distancing among outpatients and staff. This model of rapid implementation of telerehabilitation allowed us to continue to provide access to care while maintaining patient satisfaction.

There were several key elements of implementation. First, there was consistent communication among stakeholders within and between departments. Open forums for discussion and feedback ensured that we were rolling out a complete product. Secondly, leveraging existing resources created innovative supports for implementation of a new service delivery model for meaningful care. We were able to leverage our evidence-based practice, research, and quality improvement team members to quickly synthesize and disseminate information about best practices via telerehabilitation. Additionally, our scheduling team and administrative supports took an active role in educating our families on how to set up their technology for the best experience prior to that first video visit. Lastly, key partnerships within other departments of the hospital allowed us to quickly share resources amongst leadership teams to anticipate frequently asked questions and identify common barriers and their already established solutions. Because of these relationships, the rapid model of telerehabilitation implementation was a successful experience for both staff and patients.

While the best option for many patients will be to return to in-person visits once the pandemic is over, specific populations may benefit from continued access to telerehabilitation visits (Ben-Pazi et al., 2020). One of these populations is those working on generalization of skills learned in clinic to home (e.g., patients with ASD, those recovering from an injury; Tenforde et al., 2017) Often the last phase of motor learning is practice in other environments (Schmidt & Lee, 2005). Leveraging telerehabilitation in this way allows a therapist to continue to provide coaching through the next phase of learning in an environment where the child needs to perfect those skills. Telerehabilitation may be a useful adjunct service for consultation in between in-person clinic visits, either for those in rural areas who may travel a long way to come to clinic or as a means of assessing the fit and function of equipment that cannot be brought into a clinic (e.g., a stander). Finally, telerehabilitation may be a feasible model for servicing medically fragile or immunocompromised children who cannot safely access therapy services in their home or community.

It is important to acknowledge that, while we were ultimately able to retain 73.5% of our patient volume, a presumed 26.5% of patients did not receive care during this time. Anecdotally, some patient families electively paused services, taking advantage of this natural break in therapies while they set up new daily structures for themselves. For patients in some departments, this was not a choice; in fact, some of the requirements that made our rapid implementation possible actually excluded specific patient populations, including bilingual families and those without Internet or phone access (although there were public programs to provide telecommunication access during the COVID-19 pandemic). This is consistent with research that language and technology access are significant barriers to telerehabilitation (Brophy, 2017; Utidjian & Abramson, 2016). In our efforts to maintain connections with our patients and families, for those with limited access to technologies or language barriers, a telerehabilitation phone call was utilized. Throughout the rapid implementation of telerehabilitation, we were forced to recognize that social determinants of health play a role in access to care, and specifically with this modality that requires a technology element. As a hospital and Division of Clinical Therapies, we are committed to health equity and have worked to further expand access to this model of care.

## LIMITATIONS

There are several limitations to this paper. This data was gathered as part of our ongoing efforts in quality improvement and during a real-time public health crisis; therefore, there is lack of experimental control and unstandardized methods were used for surveying patient families. There were no control groups.

## CONCLUSIONS

In conclusion, pediatric rehabilitation is feasible to provide through telerehabilitation technologies and families report high levels of satisfaction with this model of care. Further research needs to be completed on this service delivery model and its impact on patient outcomes. Additionally, future work is needed to refine and expand access to these telerehabilitation technologies. While continued access to telerehabilitation will not replace in-person care, it is an important adjunct for therapies to provide access to meaningful care and offer innovative treatment approaches.

## References

[R1] American Occupational Therapy Association. (2018). Telehealth in occupational therapy. *American Journal of Occupational Therapy*, 72(Supplement_2), 7212410059p1–7212410059p18. 10.5014/ajot.2018.72S219 30674404

[R2] American Physical Therapy Association. (2019). *Position on telehealth*. American Physical Therapy Association https://www.apta.org/apta-and-you/leadership-and-governance/policies/telehealth

[R3] American Speech-Language-Hearing Association. (2020). *Telepractice*. https://www.asha.org/PRPSpecificTopic.aspx?folderid=8589934956&section=Overview

[R4] Badawy S. M., & Radovic A. (2020). Digital approaches to remote pediatric health care delivery during the COVID-19 pandemic: Existing evidence and a call for further research. *JMIR Pediatrics and Parenting*, 3(1), e20049 10.2196/20049 32540841 PMC7318926

[R5] Ben-Pazi H., Beni-Adani L., & Lamdan R. (2020). Accelerating telemedicine for cerebral palsy during the COVID-19 pandemic and beyond. *Frontiers in Neurology*, 11, 746 10.3389/fneur.2020.00746 PMC7332840 32670193

[R6] Brennan D. M., Georgeadis A. C., Baron C. R., & Barker L. M. (2004). The effect of videoconference-based telerehabilitation on story retelling performance by brain-injured subjects and its implications for remote speech-language therapy. *Telemedicine Journal and E-Health*, 10(2), 147–154. 10.1089/tmj.2004.10.147 15319044

[R7] Brophy P. D. (2017). Overview on the challenges and benefits of using telehealth tools in a pediatric population. *Advances in Chronic Kidney Disease*, 24(1), 17–21. 10.1053/j.ackd.2016.12.003 28224938

[R8] Burke B. L., Hall R. W., & The Section on Telehealth Care (2015). Telemedicine: Pediatric applications. *Pediatrics*, 136(1), e293–e308. 10.1542/peds.2015-1517 26122813 PMC5754191

[R9] Cason J. (2014). Telehealth: A rapidly developing service delivery model for occupational therapy. *International Journal of Telerehabilitation*, 6(1), 29–35. 10.5195/ijt.2014.6148 25945220 PMC4352999

[R10] Coyle J. (2012). Tele-dysphagia management: An opportunity for prevention, cost-savings and advanced training. *International Journal of Telerehabilitation*, 4(1), 37–40. 10.5195/IJT.2012.6093 25945196 PMC4296812

[R11] Crutchley S., & Campbell M. (2010). Telespeech therapy pilot project: Stakeholder satisfaction. *International Journal of Telerehabilitation*, 2(1), 23–30. 10.5195/ijt.2010.6049 25945170 PMC4296786

[R12] Dorsey E. R., & Topol E. J. (2016). State of telehealth. *The New England Journal of Medicine*, 375(2), 154–161. 10.1056/NEJMra1601705 27410924

[R13] Douglas N. F., Hinckley J. J., Haley W. E., Andel R., Chisolm T. H., & Eddins A. C. (2014). Perceptions of speech-language pathologists linked to evidence-based practice use in skilled nursing facilities. *American Journal of Speech-Language Pathology*, 23(4), 612–624. 10.1044/2014_AJSLP-13-0139 24989317

[R14] Eckberg Zylstra S. (2013). Evidence for the use of telehealth in pediatric occupational therapy. *Journal of Occupational Therapy, Schools, & Early Intervention*, 6(4), 326–355. 10.1080/19411243.2013.860765

[R15] Gloff N. E., LeNoue S. R., Novins D. K., & Myers K. (2015). Telemental health for children and adolescents. *International Review of Psychiatry*, 27(6), 513–524. 10.3109/09540261.2015.1086322 26540584

[R16] Hall N., Boisvert M., & Steele R. (2013). Telepractice in the assessment and treatment of individuals with aphasia: A systematic review. *International Journal of Telerehabilitation*, 5(1), 27–38. 10.5195/ijt.2013.6119 25945211 PMC4296832

[R17] Hill A. J., Theodoros D. G., Russell T. G., Cahill L. M., Ward E. C., & Clark K. M. (2006). An internet-based telerehabilitation system for the assessment of motor speech disorders: A pilot study. *American Journal of Speech-Language Pathology*, 15(1), 45–56. 10.1044/1058-0360(2006/006) 16533092

[R18] Lee A. C., Davenport T. E., & Randall K. (2018). Telehealth physical therapy in musculoskeletal practice. *The Journal of Orthopaedic and Sports Physical Therapy*, 48(10), 736–739. 10.2519/jospt.2018.0613 30270782

[R19] Little L. M., Pope E., Wallisch A., & Dunn W. (2018). Occupation-based coaching by means of telehealth for families of young children with autism spectrum disorder. *American Journal of Occupational Therapy*, 72(2), 7202205020p1–7202205020p7. 10.5014/ajot.2018.024786 29426380

[R20] Lovo Grona S., Bath B., Bustamante L., & Mendez I. (2016). Case report: Using a remote presence robot to improve access to physical therapy for people with chronic back disorders in an underserved community. *Physiotherapy Canada*, 69(1), 14–19. 10.3138/ptc.2015-77 PMC5280046 28154440

[R21] McGill M., Noureal N., & Siegel J. (2018). Telepractice treatment of stuttering: A systematic review. *Telemedicine and E-Health*, 25(5), 359–368. 10.1089/tmj.2017.0319 30063187

[R22] Nelson E.-L., & Sharp S. (2016). A review of pediatric telemental health. *Pediatric Clinics of North America*, 63(5), 913–931. 10.1016/j.pcl.2016.06.011 27565368

[R23] Ohannessian R., Duong T. A., & Odone A. (2020). Global telemedicine implementation and integration within health systems to fight the COVID-19 pandemic: A call to action. *JMIR Public Health and Surveillance*, 6(2), e18810 10.2196/18810 32238336 PMC7124951

[R24] Olayiwola J. N., Magaña C., Harmon A., Nair S., Esposito E., Harsh C., Forrest L. A., & Wexler R. (2020). Telehealth as a bright spot of the COVID-19 pandemic: Recommendations from the virtual frontlines (“frontweb”). *JMIR Public Health and Surveillance*, 6(2), e19045 10.2196/19045 32479413 PMC7318864

[R25] Olson C. A., McSwain S. D., Curfman A. L., & Chuo J. (2018). The current pediatric telehealth landscape. *Pediatrics*, 141(3). 10.1542/peds.2017-2334 29487164

[R26] Rangarathnam B., Gilroy H., & McCullough G. H. (2016). Do patients treated for voice therapy with telepractice show similar changes in voice outcome measures as patients treated face-to-face? *EBP Briefs*, 11(5), 1–6.

[R27] Reifenberg G., Gabrosek G., Tanner K., Harpster K., Proffitt R., & Persch A. (2017). Feasibility of pediatric game-based neurorehabilitation using telehealth technologies: A case report. *American Journal of Occupational Therapy*, 71(3), 7103190040p1–7103190040p8. 10.5014/ajot.2017.024976 28422630

[R28] Sackett D. L., Rosenberg W. M., Gray J. A., Haynes R. B., & Richardson W. S. (1996). Evidence based medicine: What it is and what it isn't. *BMJ (Clinical Research Ed.)*, 312(7023), 71–72. 10.1136/bmj.312.7023.71 PMC2349778 8555924

[R29] Sauers-Ford H. S., Hamline M. Y., Gosdin M. M., Kair L. R., Weinberg G. M., Marcin J. P., & Rosenthal J. L. (2019). Acceptability, usability, and effectiveness: A qualitative study evaluating a pediatric telemedicine program. *Academic Emergency Medicine: Official Journal of the Society for Academic Emergency Medicine*, 26(9), 1022–1033. 10.1111/acem.13763 30974004 PMC6732030

[R30] Schmidt R. A., & Lee T. D. (2005). *Motor control and learning: A behavioral emphasis, 4th ed* (pp. vi, 535). Human Kinetics.

[R31] Shigekawa E., Fix M., Corbett G., Roby D. H., & Coffman J. (2018). The current state of telehealth evidence: A rapid review. *Health Affairs*, 37(12), 1975–1982. 10.1377/hlthaff.2018.05132 30633674

[R32] Sutherland R., Trembath D., & Roberts J. (2018). Telehealth and autism: A systematic search and review of the literature. *International Journal of Speech-Language Pathology*, 20(3), 324–336. 10.1080/17549507.2018.1465123 29709201

[R33] Tenforde A. S., Hefner J. E., Kodish-Wachs J. E., Iaccarino M. A., & Paganoni S. (2017). Telehealth in physical medicine and rehabilitation: A narrative review. *PM & R: The Journal of Injury, Function, and Rehabilitation*, 9(5S), S51–S58. 10.1016/j.pmrj.2017.02.013 28527504

[R34] Tomines A. (2019). Pediatric telehealth: Approaches by specialty and implications for general pediatric care. *Advances in Pediatrics*, 66, 55–85. 10.1016/j.yapd.2019.04.005 31230700

[R35] Utidjian L., & Abramson E. (2016). Pediatric telehealth: Opportunities and challenges. *Pediatric Clinics of North America*, 63(2), 367–378. 10.1016/j.pcl.2015.11.006 27017042

[R36] Weidner K. & Lowman J. (2020). Telepractice for adult speech-language pathology services: A systematic review. *Perspectives of the ASHA Special Interest Groups*, 5(1), 326–338. 10.1044/2019_PERSP-19-00146

[R37] Zoom. (2020). *Zoom Video Communications Inc*. https://zoom.us/docs/doc/Zoom-Security-White-Paper.pdf

